# Senior Managers’ Experience with Health, Happiness, and Motivation in Hospitals and the Perceived Impact on Health Systems: The Case of Meru County, Kenya

**DOI:** 10.3390/healthcare9030350

**Published:** 2021-03-18

**Authors:** Rose Nabi Deborah Karimi Muthuri, Flavia Senkubuge, Charles Hongoro

**Affiliations:** 1 School of Health Systems and Public Health (SHSPH), Faculty of Health Sciences, University of Pretoria, Pretoria 0028, South Africa; flavia.senkubuge@up.ac.za (F.S.); chongoro@hsrc.ac.za (C.H.); 2 Developmental, Capable and Ethical State Division, Human Sciences Research Council (HSRC), Pretoria 0001, South Africa; 3 Faculty of Science, Tshwane University of Technology, Pretoria 0183, South Africa; 4 Faculty of Health Sciences, University of Fort Hare, Alice 5700, South Africa

**Keywords:** happiness, health, motivation, senior managers, hospitals, healthcare workers, health workforce, health systems, qualitative research, Kenya

## Abstract

Hospitals play a significant role in health systems. Studies among the health workforce have revealed their experiences with mental health challenges. In comparison, there is limited literature on their positive mental health. The purpose of this study was to explore senior managers’ experiences with health status, happiness, and motivation in hospitals and the perceived impact on the health system in Kenya. This qualitative study applied a phenomenological research design. Senior managers within the hospital management teams were selected using purposive sampling. Semi-structured interviews were carried out among senior managers across eleven hospitals in Meru County, Kenya. Among the eleven participants 63.6% were female and 36.4%, were male and the mean age was 44.5 years. The audio-taped data were transcribed and analyzed using Colaizzi’s phenomenological approach. The five themes revealed were: (1) Happiness in the health system; (2) Health status in the health system; (3) Motivation in the health system; (4) Challenges in the health system; (5) Possible solutions to the challenges in the health system. This study revealed the positive and negative impact of the three domains, challenges, and solutions, from the senior managers’ perspective. Healthy, happy, and motivated senior managers and healthcare workers are more responsive and perform better. Policy interventions and programs promoting happiness, health status, and motivation are necessary for strengthening the health workforce and health system.

## 1. Introduction

A health system consists of all actors, institutions, and resources that handle health actions geared towards promoting, maintaining, and restoring health within the population [1]. Hospitals are a significant component in building a robust health system [2]. Hospitals employ healthcare workers who deliver services towards improving health at the community and national levels [3]. The hospital organogram in Kenya has three categories: senior managers, mid-level managers, and front-line workers [4]. According to the World Health Organization (WHO), health workers are at the heart of each advancement within a health system [5]. Health workers are individuals whose main aim is to promote and restore people’s health [5]. Health workers influence and perform health actions across all levels of any health system. However, severe shortage of healthcare workers is a major problem in Kenya [5].

Kenya is a resource limited setting with various mental health issues, which signify the need for psychosocial support [6]. In the Kenyan context, majority of mental health related studies among healthcare workers revealed evidence of various psychosocial risk factors in the workplace including anxiety [7], burnout [8,9,10], depression [7], chronic stress [9], and substance abuse [11]. Psychosocial risk factors in the workplace “are among the leading causes of early retirement from work, high absence rates, overall health impairment, and low organizational productivity.” [12]. In the health systems context, the effects of these mental health challenges negatively impact the well-being of healthcare workers, reduce the quality of care, and result in underperformance [9]. Thus, it is paramount to have evidence based prevention interventions [9]. Seligman et al. believe that more studies focusing on identifying and amplifying people’s strengths are necessary and as important as studies on mental illness [13]. The adverse effects of mental health issues signify the importance of action-based research which enhance our understanding of various concepts while proposing solutions is equally important.

However, in Kenya there is a dearth of research on mental health aspects among healthcare workers. Thus far, studies that have focused on healthcare workers well-being factors have used a qualitative approach to evaluating this relatively novel area. Happiness, health, and motivation are three phenomena that are related to mental health, which have been evaluated mostly through quantitative approaches. Quantitative studies in the Kenya assessing healthcare workers’ happiness [14], health-related quality of life [15], and motivation [16,17,18,19] have also been published. In 2020, systematic reviews on healthcare workers happiness globally [20] and motivation in East Africa [21] were also published. These studies and reviews revealed an existing literature gap in healthcare workers’ experience of happiness, health, and motivation in the context of health systems, especially in the African region [15,16,20,21]. Many of these studies measured these phenomena quantitatively, and hitherto regarded as more objective and reliable because of the numeric data [22]. However, researchers in health-related disciplines are now embracing both quantitative and qualitative research methods, to generate interventions and inform future hypotheses [22].

In comparison to quantitative studies, there is also a dearth of qualitative research on the topic understudy. A qualitative approach would enable an in-depth understanding of what it means for a health system to have a happy, healthy, and motivated health workforce. To bridge this knowledge gap, we aimed to explore hospital senior managers’ experiences with happiness, health, and motivation in the in the context of health system strengthening in Kenya. We believed senior hospital managers who are mostly medically trained, would provide more in-depth insight, as practicing healthcare workers, regarding the role of happiness, health, and motivation in health service delivery and productivity within a health facility and the health system. Senior managers are also referred to as the Hospital Management Team (HMT) and include the medical superintendent, nursing officer-in-charge, and the health administrative officer [4].

The HMT is in-charge of the operations that facilitate best practice within the hospital and include implementing policies, overseeing transformations, and ensuring effective performance [3]. The role of the medical superintendent is communication between policymakers and healthcare workers and vice versa, overseeing all the administrative functions, and leading health services providers such as physicians, nurses, pharmacists, and all allied services within the hospital [4]. The nurse in-charge plans, monitors, and coordinates the administrative and clinical services of nurses under their supervision, while facilitating the patient care services. The health administration officer is in-charge of planning, budgeting, and advocating for resources needed in the hospital [4]. In Kenya, senior managers such as medical superintendents and nurses in charge are medically trained and have clinical experience. Therefore, they represent the management and front-line health workers.

Using a qualitative approach, this study explores senior managers’ experiences with health status, happiness, and motivation in hospitals and the perceived impact on the health system in Kenya. Currently there is no universal definition of happiness, health status, and motivation. In this study, a happy person is one that experiences positive emotions and intrinsically perceives their life as meaningful and valuable [20,23]. Health status in this study, is viewed as a multifaceted concept that entails an individuals’ evaluation of their physical, mental, and social well-being [15,24,25]. Motivation is the driving force one has, to achieve a goal such as the health workforce and health system responsiveness [21,26,27]. This qualitative study will contribute to the existing literature and inform health policymakers and stakeholders in developing evidence-based mental health promotion policies related to healthcare workers’ health status, happiness, and motivation.

## 2. Materials and Methods

### 2.1. Study Setting

In Kenya, the rural and remote areas are home to about 70% of the national population and have the greatest challenge of shortage of healthcare workers, with more healthcare workers in urban areas [28]. We did the present study in public and mission hospitals in a rural setting, known as Meru County, in Kenya’s Republic. The focus was on Tier 3 health facilities, including primary care hospitals (level 4) and secondary care hospitals (level 5). Tier 3 hospitals are at the top of the county referral healthcare system and in charge of the health facilities that offer lower levels of care such as a Tier 1 offering community care (level 1) and, Tier 2 offering primary care facilities (level 2 and 3) [28]. Specifically, the study was done in eleven hospitals, both public and mission, between 7 July and 11 July 2020. In Kenya’s rural areas, hospital ownership is majorly by government and faith-based organizations (mission). In Kenya, rural areas have more mission hospitals than private-for-profit health facilities and admit up to 17.9% of patients [28]. Thus, we conducted in-depth interviews among senior managers in public and mission hospitals. We did the study during the global Coronavirus disease (COVID-19) pandemic. During the fieldwork phase, Meru County had recorded only thirty-two COVID-19 cases [29].

### 2.2. Study Design

This research followed the ‘Standards for Reporting Qualitative Research: A Synthesis of Recommendations (SRQR)’ guidelines [30]. In this study, the phenomenological approach was applied to answer the research question. Phenomenology is a type of qualitative research that explores and interprets a person’s lived experiences within the world, regarding a concept or phenomenon [31]. Through phenomenological inquiry, researchers can understand an individuals’ in-depth qualitative description and meaning in consciousness regarding their experience of phenomena [32]. Using phenomenology inquiry, specifically through exploratory semi-structured in-depth interviews, we explored healthcare managers’ understanding of concepts of happiness, health status, motivation, and the perceived impact on the Kenyan health system. Exploratory semi-structured interviews aim at exploring the participants experiences with phenomena or issues from diverse viewpoints [33,34]. The open-ended questions allowed participants to provide detailed information based on their thoughts, feelings, and opinions [33] related to their happiness, health, and motivation in the health system. The fact that these were relatively novel phenomena in the context of health systems resulted in the application of an exploratory approach. According to DeJonckheere, semi-structured interviews are important in health research and provided focus and flexibility through in exploring the phenomena understudy while allowing for additional probing when necessary [34].

### 2.3. Participants

Purposive sampling was used to select the participants in this study. According to Emmel, “The purpose of purposeful sampling is to select information rich cases that provide insight into the research questions and will convince the audience of the research” p. 2 [35]. Purposive sampling allows the researcher(s) to scientifically inquire about an individual’s experience using a pragmatic design [35]. Applying a pragmatic design gave us an opportunity to contribute to informed action via our research, through prioritizing credibility over certainty which is paramount when exploring relatively new phenomena or context [35]. The participant selection criteria were as follows: (1) healthcare workers who were part of the hospital management team at the hospital level; (2) minimum of one year of work and HMT experience in Kenyan public or mission hospital; (3) participants who willingly signed the informed consent form agreeing to participate in this study were included. Based on these criteria, eligible and willing participants regardless of any form of disability or special need, were included. The exclusion criteria included: (1) healthcare workers who during the time of data collection were not part of the hospital management team; or (2) had less than a year of work or HMT experience in a Kenyan public or mission hospital; (3) did not voluntarily sign the informed consent form agreeing to participate. All eligible participants were informed that participation in this study was strictly on voluntary basis following all the ethical guidelines.

Data collection was completed once saturation was evident by the ninth interview, but two more interviews were done to confirm the same. Saturation is the point at which no new themes are emerging; thus, more interviews are no longer viable [36]. According to Polkinghorne, interviews for phenomenological research should include five individuals and above [32]. The senior managers represented the HMT and healthcare workers from an in-depth perspective; thus, provided important information on the phenomena understudy [37]. Therefore, the hospital managers were the key informants in this study. No participant withdrew from this study. To ensure anonymity and confidentiality of the participants, alpha-numeric codes such as II1 to II11, where II indicates in-depth interview, were used in the data analysis and report writing instead of personal identification information such as names or identity card numbers. Among the eleven participants recruited, the mean age was 44.5 years (SD = 7.05) and 63.6% were female while 36.4%, male. Approximately 45.5% of participants were from mission hospitals and 54.5% from public hospitals (as shown in Table 1). This proportion is comparable to the hospital ownership statistics in Meru County, where among the 24 Tier 3 (level 4 and 5) hospitals 66.6% are public, and 33.4% are mission [27]. Our sample consisted of all senior managers within the Kenyan hospital organogram, namely medical superintendents, nursing officers in charge, and a health administrative officer (as shown in Table 1). The average duration of working experience among the participants was 16.61 years (SD = 7.94).

### 2.4. Data Collection

In this study, the first author (R.N.D.K.M) who is trained in qualitative research conducted the face-to-face interviews. The key informant in-depth interviews with the eleven senior hospital managers involved a semi-structured interview guide. According to the participants’ schedules and preferred location, which for all the participants happened to be their office/consultation room at the hospitals they worked in. At the beginning of the interview information on the participants’ age, sex, hospital ownership, duration of work experience, and role in the hospital management team, were obtained. When necessary probing questions were asked to enhance our understanding through detailed explanations of ideas, feelings, and opinions (see in Appendix A Table A1). The interviews were carried out in the English language, a national language, and the medium of instruction in secondary and tertiary education institutions, such as medical schools and health-related training colleges in Kenya. The interviews were audio-recorded, following both written and verbal permission from the participants. However, transcripts did not include any identifying information of the participants.

### 2.5. Data Analysis

Colaizzi’s phenomenological approach has been used in recent qualitative studies among healthcare workers such as Shahdadi et al., in 2018 [38], and Liu et al. in 2020 [39]. Thus, the Colaizzi phenomenological approach was applied [40] in the analysis, as adapted by Morrow et al. [41]. Following transcription of the audio tapes, the data analysis was done in a stepwise form following the Colaizzi phenomenological approach. Step 1: familiarization of the transcripts done by all the authors. Step 2: identification of significant statements related to the happiness, health, and motivation phenomena under study was done by RNDKM and confirmed independently by CH and FS. Step 3: formulation of meaning based on significant statements. Step 4: thematic clustering, incorporation of all themes through grouping the similar and recurring statements was done by RNDKM and confirmed by CH and FS independently after which a consensus on the final emerging theme categories and clusters was agreed on based on our data (as shown in the results section). Step 5: exhaustive description done by RNDKM and counter checked by CH and FS. Step 6: capturing the fundamental structure of the phenomena in short and dense statements. Step 7: validation of the research findings was done by all authors [41]. Disagreements were discussed and amicably resolved in meetings through discussions.

### 2.6. Ensuring Research Quality and Rigor

Trustworthiness of the study was ensured through various methods. In this study, triangulation was used as a credibility strategy. Triangulation is the process of examining research integrity by using different methods, and sources to support the results and reduce bias [42,43]. In this study, triangulation was performed through the thick description, direct quotations from interviewees’ responses, and comparison with prior similar studies. Transferability was accomplished through purposive sampling of the participants and the thick description. This study’s thick description was done by ensuring meaningful findings are captured and described in their appropriate context and detail [43]. Confirmability was achieved through audit trailing of raw data included in the audio-tape recordings, transcriptions, process notes of deconstruction, synthesis, and reconstruction using Colaizzi’s phenomenological approach. Finally, we achieved dependability by carrying out interviews to the point of thematic saturation, which was the ninth interview, after which two more were done to confirm.

### 2.7. Ethical Consideration

Ethical approval for this study’s protocol was obtained from the Research Ethics Committee, Faculty of Health Sciences, at the University of Pretoria, South Africa (approval number: 718/2019). In Kenya, the ethical approval was obtained from the Institutional Review Board (IRB), United States International University-Africa (approval number: USIU-A/IRB/130-2020). To fulfil the Kenyan research ethical requirements, ethical approval from an Institutional Review Board in Kenya had to be obtained prior to applying for a national research license. Following ethical approval from the IRB, a national research license (reference number: 901924 and license number: NACOSTI/P/20/4133) was obtained from the National Commission for Science, Technology, and Innovation (NACOSTI), Kenya. All participants provided both verbal and written informed consent.

## 3. Results

A total of five themes categories and twelve and sub-themes (clusters) based on our sample of 11 senior managers (as shown in Table 2).

### 3.1. Theme 1: Happiness in the Health System

During the interviews, participants associated happiness with high responsiveness, good performance, high-quality healthcare service delivery, being oneself, being happy at work and home, and satisfied patients. The participants describe the positive impact of being happy, and the negative impact of unhappiness as:

“Because when you are happy at work, even at home you will be happy… Then, a happy staff will give good results or good performance. But if I am not happy, I will not perform.”

(II1)

“So, the clients will be moving up and are happy and from that hospital they will be shining, and they will be happy.”

(II6)

“…the joy of every health worker, whereby you can be able to see a patient and help them to the full capacity…Yes, of course. If I am not happy at work, then it means I am not going to work well. I am not going to be myself, in helping the patient, so it will affect because I will not be able to give the patient enough care that he or she needs at that particular time.”

(II8)

#### 3.1.1. Factors of Happiness in the Health System

These are the factors that could contribute to the participants’ happiness working in the health system: good leadership, adequate remuneration, serving and helping patients, proper infrastructure, and resources such as medical equipment, enough medication, having good relationships with co-workers, sense of achievements such as satisfied patients and appreciation, which participants described as:

“Those are the things, if we work as a team, we appreciate one another, one will work better… It will because when we come on duty, working as a team we will have no problem and… our patients and the clients will be happy when they leave the hospital.”

(II3)

“To see that my clients are happy and satisfied and to have all that I need to offer the services. And maybe the measures are there to prevent maybe things like accidents occurring in the workplace, that my… that we are okay with other staff.”

(II9)

“For sure one of the factors, is when our patients are happy about our service, this one is the feedback that we can get for sure. Then being a mission hospital, our mission is really to target the poorest amongst the poor.”

(II11)

#### 3.1.2. Factors of Unhappiness in the Health System

The participants also reported factors that contribute to their unhappiness. The elements of unhappiness included low remuneration, continuously delayed pay, feelings of oppression, work environment, work overload, career stagnation, and lack of medical resources described as:

“Yes, there are, like remuneration, salary, like if the salary is delayed it can also hinder that service provider from giving quality services. The other factor is, if they are not promoted, you see this promotion is from the employer. If the employer is not promoting you, you are just stagnated in one place for a long time, you also won’t be able to give quality services that is needed. That comes around with you know, the money, you see if the salary you are being paid is not commensurate with the services you are giving, you will also not be able to give you proper services and this will cascade down to the family level… If they are not happy, you are also not happy. So, you see you get a mental problem, you will not be able to give your quality services.”

(II5)

“For example, if I am able to work in a suitable environment whereby, I am not under pressure, I am comfortable the hours are not… I am not oppressed, like I am not over working, yes. I have the right materials that are needed for me to work and maybe to treat my patients.”

(II8)

### 3.2. Theme 2: Health Status in the Health System

Participants in this study reported the importance of healthcare workers’ health status in the health system. Good health contributed to good performance, quality health service delivery, productivity, mental sobriety, and reduced wastage. In contrast, poor health was associated with low or no productivity and low quality of care. The participants described the positive impact of being healthy and the negative impact of working with poor health as:

“The health, is very important, because once one is healthy, they are able to accord proper, or good, or quality services to the client who comes in. They are able to be sober; their minds are not disturbed and hence good output in terms of productivity in the facility and also for their comfortability. There is no wastage of time in the hospitals, there is no wastage of time and socials of moving up and down, so they are efficient, and they are effective at the place they are allocated.”

(II6)

#### 3.2.1. Health Status Factors in the Health System

The factors that contributed to senior managers’ health were health insurance cover such as the National Health Insurance Fund (NHIF) and Saham covering them and their nuclear family. Other health-promoting factors are safe working conditions, adequate remuneration, adequate infrastructure, funding, adequate personal protective equipment, comfortability at work, good workplace relationships with staff and patients, support from supervisors, and government, described as:

“The factors which affect my health… in adequate infrastructures, in adequate funding, yeah that is some of the factors which mostly affect my working conditions.”

(II4)

“Infection prevention, I’d say maybe the relationship between… with other staff, the relationship with the clients, if I am comfortable at work, I know I am healthy.”

(II9)

“The work environment that you work, the remuneration, also any other support that we get from our employer and our supervisors, either at the sub-county or the county level.”

(II10)

#### 3.2.2. Factors Contributing to Poor Health Status in the Health System

Poor health among the participants was due to delayed salaries, poor hygiene, lack of infection prevention mechanisms, lack of resources, a severe shortage of healthcare workers leading to physical strain and injury, and knowledge gaps, described as:

“Okay if the general hygiene of the hospital is not good it can affect especially on the side of infection prevention. Of course, human resource is never enough so when we have very few workers, those ones who are trained, strain a lot and we end up getting people with back problems due to over working or wrong standing. So, at the end of it or at old age you start complaining of back ache, joint pains.”

(II1)

“There are several factors, one is either sickness. You know the staff can fall sick while they are at the hospital, some have disability, you can have either collapse of the disc because of lifting the patient, it is also a factor. Another factor is availability of the necessary equipment, for the day to day running of the facility or shortages of either commodities like drugs, we have shortage.”

(II5)

“And also, the capacity of the or the numbers of the health workers it also affects, because you find we are understaffed and so sometimes it is hard for all the health workers to work, so it affects them, one way or another.”

(II8)

### 3.3. Theme 3: Health Worker Motivation in the Health System

Motivated senior managers were more likely to motivate each other, provide quality care, perform highly, manage effectively, provide knowledge to patients, avoid fraudulent behaviors, and have satisfied patients. Lack of healthcare workers’ motivation negatively impacts the health system in similar ways like longer waiting times, patient complaints, and loss of confidence in health facilities, which affect health service delivery. Participants’ described this as:

“Okay motivation, is very important… If you are motivated, you also perform well. And motivated staff are like a hungry lion, so when you come into contact with a patient and you are not happy, you might end up even mishandling that patient, unknowingly because you are even stressed up.”

(II1)

“And once the staff are motivated in their work it becomes very easy in management… So, they will be efficient, and they will not be found with a patient, with so many frauds, like they cannot be able to make ends meet together with the client. So, they are able to solve their problems, they are able to analyze the problem, they are able to make diagnosis even through the assessment and talking with the client, they are able to make the assessment even with the counselling and a bit of skills.”

(II6)

“People have had the motivation to help the patients, you know that one motivates you because this is what brought you here, this is why you are working in the first place. Yeah, so it is a big motivation… I am not motivated to work that day obviously; I will not be so eager to see the patients. I will just be there, maybe could be rude to patients.”

(II8)

#### Motivating and Demotivating Factors in the Health System

Motivating factors reported by participants were enough remuneration and allowances, non-discriminative pay, sense of achievement, positive client feedback, religious dedication, helping patients, empathy, a unified vision, teamwork, quality medical commodities, and staff accommodation. Regardless of the multiple challenges, some participants believed being a health professional was a calling. The demotivating factors were the opposite of motivating factors such as the lack of equipment, bad working environment, and low customer satisfaction. The participants described this as:

“Good remuneration, good health sector at least allowances when we are being paid and, non-discrimination of paying allowances to health workers. Because there are some cadres which do not receive some benefits and allowances, I think if that is worked on it can motivate those other staff members.”

(II4)

“First of all, as a healthcare professional, it’s like a calling. From my observation, when the healthcare workers are not motivated, okay they tend not to give the best services. Maybe a customer or a client is delayed more, or they are not served at the right time, or does not get the right service. Simply because the healthcare worker is not motivated or is not working in the right environment.”

(II10)

### 3.4. Theme 4: Challenges in the Health System

During the interviews, the senior managers reported some challenges they experienced working in the Kenyan health system. These challenges hindered the participants’ ability as trained healthcare workers to provide quality care to their patients and be happy, healthy, and motivated. Thus, resulting in the suboptimal functionality of the healthcare system. A severe shortage of healthcare workers, poor planning, low and delayed salaries, lack of necessary medical equipment and supplies, poor work conditions, favoritism and delays, and limitations of the NHIF are challenges reported by the participants.

#### 3.4.1. Low and Delayed Salaries

The senior managers and health workers in the Kenyan health system experienced challenges related to delayed pay (at times for months in government owned health facilities), and low and non-competitive remuneration. The unpredictability in the timing of payments resulted in some healthcare workers venturing, where possible, in alternative income-earning activities for survival. The following quotes capture the current situation:

“Unless, maybe for the last three months, just the delays of salaries.”

(II2)

“Another thing is probably in the public sector is, the remuneration is not that good, it is not competitive… But you see the ones who are on contract are better remunerated than those who are on permanent and pensionable.”

(II7)

“It is not a secret that, many of the government health workers, they have a second job, a private clinic or something like that. This is clear to everybody it is not a secret.”

(II11)

#### 3.4.2. Severe Shortage of Healthcare Workers and Lack of Support

Most of the participants reported a severe shortage of healthcare workers. Some stated their facilities were understaffed, and there was a high turnover of healthcare workers. Understaffing was associated with physical issues like back and joint pain and psychological problems such as burnout and feeling like they are dying silently due to a lack of psychological support. Capacity building to bridge knowledge gaps due to minimal continuous education among the in-service healthcare workers was another challenge. The shortage of healthcare workers resulted in the current health workforce performing tasks beyond their job description.

*“I came to realize that the burnouts which come through in some of the healthcare workers, it is because nobody thinks they need help… When that healthcare provider is sick, nobody knows if that person needs any help… They are dying silently.”*

(II2)

*“And other times they need to be re-energized with some knowledge, with that continuous education, it’s not very frequent, it takes time. So, capacity building, is actually very key.”*

(II6)

*“So, most of the staff, the turnover time of most of the staff is high because of the going to other facilities to seek for better employment.”*

(II7)

*“…we’ve really had a times when there are real shortages. You find that some departments maybe do not have enough staff.”*

(II9)

#### 3.4.3. Poor Planning

Poor planning by health leaders was a challenge that adversely affected the health workforce and health service delivery. The challenges experienced included shortage of health services, lack of necessary medical equipment and essential commodities such as medication, delays in delivery of supplies, and poor infrastructure that is below hospital level requirements. Poor planning resulted in delayed responsiveness, congestion of patients, patient complaints, continuous and avoidable referral of patients. However, inconsistencies of facilities and supplies were reported, with some facilities receiving supplies and others not receiving, thus causing disagreements between staff and higher-level management in government, described as:

“Poor planning in the sense that…what you use to serve the clients, where it is medicine, treatment, sometimes you may not be able to get the supplies in good time. Others may be in certain facilities; others are not in certain facilities. Like sometimes in public hospitals, there may be strikes, probably the management and staff are not agreeing, so there may be a strike that would cripple the services and then this reflects badly on the hospital, the hospital staff, and the government.”

(II7)

“You have no theatre, you have no inpatient, it’s challenging because now you can’t give your full attention to this patient even if you are able to, because there are no facilities that are needed in that particular area… And also, sometimes you find the facilities that are needed like now the drugs, there is the delay of supply, so sometimes you find a patient will come and go without medication, so that’s another challenge… when they come many times and they are not getting the medication they start wavering. They say, “The hospital doesn’t have this… doesn’t have that…” so that flow also, is cut by the patients.”

(II8)

“I think the two major challenges I can say are the referrals because not all the services are available at the county level. And the second one for sure is the blood, scarcity of blood.”

(II11)

#### 3.4.4. Delays and Limitations Related to the NHIF

Limitations and delays of payment for patients by the NHIF was another challenge. This challenge resulted in frustration among patients from lack of treatment due to the NHIF refusing to pay for services even though it was a compulsory national insurance fund, described as:

“Number one is the NHIF, it is quite challenging because they do not pay in time. Most of the patients are NHIF covered. Just imagine if you treat a patient for six months or one year without being paid, how are you going to manage the whole situation? Then for the patient we normally experience challenges because they do not understand how the system works. They expect when they come with the NHIF cover, you give them the medication they require, but there is limitation from the NHIF and when you are managing your patient, sometimes you find that what the NHIF has given out cannot manage this particular case.”

(II1)

### 3.5. Theme 5: Possible Solutions to Challenges in The Health System

#### 3.5.1. Health, Happiness, and Motivation Policy

Senior managers unanimously agreed to the development of policies and institutionalization of health, happiness, and motivation programs. The reason being that the three domains were perceived as very important for the health workforce and health system. An association between healthcare workers’ happiness, health, and motivation was reported by the senior managers. The participants stated that healthier, happier, and motivated healthcare workers would result in better healthcare delivery due to better teamwork, psychological sobriety, and better responsiveness thus shorter waiting times for patients, guaranteed confidentiality, friendliness/receptiveness, cleanliness, thus better patient outcomes, described as:

“If you are healthy and motivated at work, you are able to work better. In performance, when you are motivated and you have good health at work, you will be able to attend to your patients as they come, so this will reduce the waiting time… even with a settled and sober mind at work one will be able to work without biasness and even the confidentiality of the patients will be facilitated, the place where you are because you are already healthy, you are motivated, you be able to work better, it will be clean, whatever is needed for you patients to feel comfortable, you will be able to give it freely, and happily.”

(II3)

“Okay if, a healthcare worker has good health, is motivated and there is happiness, the turnaround time for patients in their service, will be quick.”

(II7)

Senior managers also stated that they were not aware of any happiness and motivation policies and programs. They believed that the government should develop and institutionalize happiness and motivation policies and programs to strengthen the health workforce which was described as:

“I think the ministry of health needs to have this policy, so that we can be able… they need to develop policy and implement them so that now we can at least be motivated.”

(II3)

Providing psychological support such as counseling, team building events, an annual increase of remunerations, harmonized salaries, and shared events for the exchange of ideas between healthcare workers in private and public hospitals were possible solutions for promoting healthcare workers’ happiness, health, and motivation. It was clear that senior managers were motivated by being involved in the process of policy development and implementation. The participants described this as:

“Yes, public, private, mission, where they can be coming together and share. Sometimes, we find that there are protocols in the government, but they never reach here. So, it’s like sometimes we are in the dark. So, if there could be something like that, people could be coming together sharing and it can help.”

(II1)

“… we decided to have a welfare, which we have at least once a year. We just get out to do team building, just being outside the facility, with just the staff. Mostly psychological…because…if you see them, they are strong but deep inside they are not ok. So, they need mostly, psychological. And this psychological can be done in terms of having those sessions of motivational speakers, having time outs, yeah something like that… I would say currently we I think it is in March this year… the most encouraging thing was those little cadres those people, if they were nurses, they were called upon to develop the policy then forward to the county. So that was the motivating thing because we were able to assign each cadre, from down coming up not upcoming down.”

(II2)

“Yes, I think going forward, foundations should be put in place… be rooted. So that the healthcare worker gets to benefit from the government by creation of institutions to improve their motivation, their health, their happiness. Either institutions like counselling, institutions like harmonized salaries, that will improve their motivation, facilities like yearly increments on salary, I think such policies would assist to make sure that the health worker remains on top and thus, motivated and with good health and I think it will reflect on their services, to their clients.”

(II7)

#### 3.5.2. Supporting the Current Healthcare Workers

The government can solve the challenges in the health system by actively supporting the current workforce. Specifically, by ensuring timely payment of salaries, competitive pay, incentives, increasing the number of healthcare workers, provide staff housing, equity of benefits, seconding the mission hospitals, continuous supervision, and frequent in-service training. The government should consider motivating healthcare workers in multiple ways through monetary incentives and ethical motivation. By supporting the healthcare workers in these ways, the quality of care and health outcomes would improve. Participants described this as:

“If the government would be assisting the faith-based hospitals and other private ones by seconding the staff. They can really help a lot.”

(II1)

“… you can have proper housing for the staff because some of their staff are staff who have to travel from very far. And then adequate meals for the services to be given.”

(II5)

“There are a lot of disparities within the health workers cadres, among the biggest cadres. I wish there could be some equity. Yes, that would motivate health workers a lot.”

(II10)

“We have, according to me, one of the weak points in the government, is that they think that just money is the only motivation for the health workers. But according to me it does not work well. They have to focus on other motivators, especially, ethical motivation, something like that.”

(II11)

#### 3.5.3. Proper Planning and Improvement of Health Facilities

Proper planning and timely execution are essential to improve the coordination between the hospitals and the national and county governments. Participants suggested the Kenyan government needs to ensure all hospitals are up to the respective standards. Raising the current standards can be done by improving the infrastructure, the timely delivery of medical supplies based on the health information sent daily, introducing a health facility financial aid during times of hardship such as a pandemic, providing timely updates, and providing welfare strategies. The facilitation of health education in communities was also encouraged by the participants. These were some methods that the interviewees suggested would improve the health system and simultaneously contribute to their happiness, health, and motivation. It was described as:

“Proper planning means the data that we collect every day, and we send to the ministry of health… this data can be used for planning purposes. When it comes to staff remuneration they should, make sure it is competitive across the board, so that they may be able to attract even the best medics in the country… And then, proper and prompt procurement processes, that do not have a lot of bottlenecks… information to the community that these services are being offered. So, if information is channeled properly through the churches, through other forums in the community or chief baraza, then most of these services can be consumed instead of staff being employed and just laying idol or the equipment laying idol with idol staff.”

(II7)

“So, if they could be able to upgrade the hospitals, and equip them with everything that is need then, I think that would be amazing, and it would really help solve the health problems around. And also, the staffing, if they are able to standardize the number of staff in each hospital.”

(II8)

## 4. Discussion

This study’s key findings show that there are common factors which cut across the senior managers’ health status, happiness, and motivation in Kenyan hospitals. Through explaining the risk and buffer factors of happiness, health, and motivation among senior managers and health workers in hospitals, the importance of health systems strengthening across all six building blocks was evident. Both the current study and previous studies have revealed that a healthier, happier, and motivated health workforce would improve the health level and health equity; improve their efficiency and responsiveness; and avoid wastage of resources, which would contribute to the attainment of the best possible patient and population health outcomes [27,44].

The first theme in this study was happiness in the health system which was divided into factors of happiness and unhappiness. The participants believe that happiness is influenced by various factors in the health system including good leadership and governance, proper human resource management such as adequate remuneration, medical altruism towards patients and co-workers, proper infrastructure, and resources such as medical equipment, good co-worker relationships, sense of achievements such as satisfied patients and appreciation. Aside from the factors related to proper leadership and adequate infrastructure and medical resources, our findings were concurrent with other qualitative studies in Iceland [45], Thailand [46], and the United States of America [47]. These studies similarly reported sense of meaning, altruism, enjoying treating their patients, professional pride, sense of personal achievement, and support were factors of healthcare workers’ happiness [45,46,47]. In the current study, the factors of senior managers’ unhappiness were majorly the opposite of all these factors plus, discrimination, career stagnation, delayed and inadequate remuneration, and inadequate medical equipment.

Health status in the health system was the second theme in this study. Senior managers in this study expressed the significance of healthy healthcare workers’ in health systems efficiency. Being physically and psychologically healthy was associated with better performance, improved quality health service delivery, and decreased wastage. In Italy, a positive relationship was reported between healthier healthcare workers and improved quality healthcare delivery to the patients they met almost on daily basis [48]. Our study revealed that, the opposite was also true with poor health which was linked to poor performance and minimal quality of care. The factors of health status revealed in this study show the importance of an enabling and supportive work environment and health system. Firstly, health insurance covers such as the NHIF and Saham had a positive impact on their health status. However, factors such as poor hygiene, lack of infection prevention equipment such as PPEs, in adequate medical resources, knowledge gaps, and shortage of health workers thus physical strain due to heavy workload, directly or indirectly negatively affected the health of senior managers and healthcare workers. Prevention of health problems is more ideal compared to cure, thus a safe, hygienic, and healthy work environment in health facilities is paramount for optimal performance and health workforce retention [15,49]. In this study, low and delayed remuneration also negatively affected their health overtly or covertly, which has previously been reported as a dissatisfier which hinders optimal performance of healthcare workers [15].

Motivation in the health system was the third theme, were motivators and demotivators were reported by the senior managers in this study. The senior managers reported motivating and demotivating factors that implied the need for improving the Kenyan health system. Participants reported that the current Kenyan health system depletes their motivation through delayed pay, lack of medical equipment, and a poor working environment. Their demotivation resulted in, patient complaints due to dissatisfaction with quality of care, and at times pushed them to engage in double practice for survival. Similar demotivating factors have been reported among healthcare workers in South Sudan [50] and Uganda [51]. The participants in this study also stated if the Kenyan health system motivated the health workforce the following would occur: more empathy, sense of achievement, teamwork, motivating colleagues, willingness to provide patients and the community with more health promotion knowledge, avoid fraudulent behaviors, and reduce the waiting time. Receiving positive feedback from patients was a motivator to continue to enjoy healthcare service provision to patients and perform better. This was similar in a study in Tanzania which reported that appreciation from the community increased health workers motivation [52]. Thus, both financial and non-financial factors would contribute to enhancing senior managers and health workers motivation in hospitals [21].

The fourth theme with most categories was the challenges in the health system faced by senior managers in the Kenyan health workforce. Multiple challenges were identified by participants including delayed and low remuneration, severe health worker shortage poor planning, and delays and limitations of NHIF for patients causing patient dissatisfaction. Previous studies in Kenya have revealed the severe health workforce shortage is due to several issues such as delayed or lack of salaries, poor infrastructure and environment, inadequate medical equipment and medication, poor human resource management, and brain drain [53,54]. These challenges are among the recurrent problems healthcare workers experience, resulting in recurrent and prolonged strikes [53,55]. This study revealed that continuously experiencing these challenges adversely affects the health status, happiness, and motivation of senior managers, health workers, and the efficiency of the health system.

For example, a challenge such as delayed and low remuneration has resulted in some healthcare workers’ moonlighting in other private health facilities to get money for themselves and their families to survive; this is due to the low and inconsistent remuneration. Researchers have reported black trade or double practice in the Kenyan health system, which means that patients are more likely to be compelled to use the out-of-pocket payment method to receive medical services, resulting a financial catastrophe for some people [56,57]. Thus impeding the chance of attaining the health system goal of protecting users from financial catastrophe when seeking healthcare services, which was the basis of NHIF in Kenya [27,58]. In 2009, Stringhini reported that receiving informal payments from patients negatively affects healthcare workers’ motivation and limits patients access to quality care [59]. This shows that engaging black trade or informal payment, and double practice is often for survival purposes but depletes motivation. Some of the challenges indicated in this study have been reported previously in African health systems such as, inadequate health workers, insufficient health finance, and poor leadership and management [60]. However, the current study confirms that these challenges are negatively impacting the responsiveness to patients non-medical expectations and are causing psychological effects among healthcare workers. To address each challenge, the senior managers suggested solutions for policy and practice.

In this study, the fifth theme was the possible solutions to the challenges being experienced by health workforce in the Kenyan health system. The solutions presented in this study, were similar to a prior study where researchers suggested ways of solving health worker shortage gaps such as competitive remuneration, improved work environment, and incentivized training of the current health workforce to effectively perform necessary tasks beyond their initial training [61]. In this study, the senior managers confirmed that the three phenomena are salient in the responsiveness to medical and non-medical patient expectations. The participants explained that when senior managers and healthcare workers are healthy, happy, and motivated; they will be more responsive and sober. This will be evident in improved performance, higher quality of healthcare delivery, educating patients on health promotion, easier management, and happier colleagues and patients. This study’s findings may have policy, practice, and research implications.

### 4.1. Implications for Policy and Practice

The present study showed the significance of systems thinking in enhancing psychosocial health at work, in the context of the Kenyan health system [62]. Specifically, by presenting qualitative findings that show the significance of promoting healthcare workers’ health status, happiness, and motivation through a holistic and intersectoral approach. Direct support from the county and national governments in conjunction with senior managers at hospitals, is important in improving the psychosocial health of healthcare workers in Kenya through addressing the factors of happiness, health status, and motivation presented in this study. The reason being most factors were at the health system level. The current study has revealed a gap in psychosocial health policy related to healthcare workers’ happiness, health status, and motivation in the Kenyan health system. By developing and institutionalizing policies and programs aimed at increasing healthcare workers’ happiness, health, and motivation, health workforce strengthening through enhancing their psychosocial health is more likely occur.

Protecting the well-being of the health workforce, will ensure avoidance and management of common psychosocial risk factors thus, prevent problems from developing at an individual, health facility, and health system level [63]. The senior managers stated that healthcare workers are dying silently, which signifies the importance of policies, programs, and plans to improve their psychosocial health. To achieve this, the Government of Kenya needs to develop psychological support through counselling, workshops, mental health training but also changing the work environment and strengthening the health system are key. The present study shows that by solving the challenges in the Kenyan health system in some ways presented here, while viewing the health workforce as core component of the health system, would improve the quality of care, patient outcomes and health indicators nationally. Our findings show that a healthier, happier, and motivated health workforce could enable the Kenyan health system to make significant progress to attaining the health system goals. Therefore, promoting the health, happiness, and motivation of the health workforce as reported by the senior managers will significantly contribute to the attainment of universal health coverage (UHC) and eventually the achievement of sustainable development goal (SDG) three on health for all at all ages [64,65]. However, the present study has limitations that provide opportunities for future studies to explore.

### 4.2. Future Areas of Research

The first limitation of the present study was that it focused on only Meru County, Kenya due to limited financial resources. Hence, future studies should be done in the remaining forty-six counties to gain nationwide perspective on similarities and/or differences in health workforce experience with happiness, health, and motivation. Secondly, this qualitative study explored three phenomena, which means the results did not measure the degree of impact. The three phenomena have been reported separately using a quantitative approach. Future studies should quantitatively measure the degree of impact, and relationship between the three phenomena in the context of health systems strengthening. In the present study, due to financial constraints, data collection was among the senior managers who were part of the HMT only, who play a role in communication between policymakers and healthcare workers. In the future, research should explore the views of various stakeholders such as health policymakers, mid-managers, and frontline health workers. Research among the different stakeholders will enlighten us on the differences and similarities in the health workers’ experience with happiness, health, and motivation, which are salient in strengthening the health system.

## 5. Conclusions

To our knowledge, this was the first study to qualitatively explore the experience of senior managers in hospitals with health status, happiness, and motivation, and the perceived impact on the Kenyan health system. This study revealed a relationship between happiness, health, and motivation and impact on the performance and well-being of senior managers. The senior managers confirmed that happy, healthy, and motivated healthcare workers are more likely to be responsive, productive, psychologically sober, and offer the highest possible quality healthcare. Based on the five themes happier, healthier, and motivated healthcare workers are likely to result in content patients and better patient and population health outcomes. This study’s key finding revealed that the factors reported cut across the three phenomena namely health status, happiness, and motivation among senior managers in hospitals. The senior managers also indicated, when healthcare workers feel less healthy, demotivated, and unhappy, the result is less responsiveness, lower quality of care, dissatisfied patients, and a poorly performing health system. Several challenges are present at the health facility and health system levels. Notably, senior managers and healthcare workers in hospitals have been resilient. This is evident through the multiple challenges they are experiencing and still significantly contribute to improving patient and population health. The research findings suggest possible solutions to the problems inhibiting health workers’ happiness, health, and motivation in the Kenyan health system. Finally, this study has revealed that involving senior managers, mid-managers, and frontline workers in health, happiness, and motivation policy development will more likely result in effective implementation and sustainable strengthening of the health workforce and health system.

## Figures and Tables

**Table 1:** Demographic characteristics of participants.

Participant Code	Hospital Ownership	Sex	Position
II1	Mission hospital	Female	Nursing officer in charge
II2	Public hospital	Female	Nursing officer in charge
II3	Public hospital	Female	Nursing officer in charge
II4	Public hospital	Male	Health administrative officer
II5	Public hospital	Male	Medical superintendent
II6	Mission hospital	Female	Nursing officer in charge
II7	Public hospital	Male	Medical superintendent
II8	Mission hospital	Female	Nursing officer in charge
II9	Mission hospital	Female	Medical superintendent
II10	Public hospital	Female	Medical superintendent
II11	Mission hospital	Male	Medical superintendent

**Table 2:** Themes and sub-themes.

Themes		Sub-Themes/Categories
Theme 1	Happiness in the health system	Factors of happiness in the health system
		Factors of happiness in the health system
Theme 2	Health status in the health system	Health status factors in the health system
		Factors contributing to poor health status in the health system
Theme 3	Health worker motivation in the health system	Motivating and demotivating factors in the health system
Theme 4	Challenges in the health system	Low and delayed salaries
		Severe shortage of healthcare workers and lack of support
		Poor planning
		Delays and limitations of related to the NHIF
Theme 5	Possible solutions to challenges in the health system	Health, happiness, and motivation policy
		Supporting the current healthcare workers
		Proper planning and improvement of health facilities

NHIF: National Health Insurance Fund.

## Data Availability

The data presented in this study are available on request from the corresponding author. The data are not publicly available due to the participants personal information.

## Appendix A. Semi-Structured Interview Guide

**Table A1:** Semi-structured interview guide.

Category	Main Questions	Possible Probe Question
Factors of health, happiness, and motivation	What factors affect your health and that of healthcare workers?	How important is the health workforce health in the health system (such as provision of quality of care and productivity)?
	What factors contribute to your happiness or that of healthcare workers?	How significant is happiness at work for you in relation to productivity and provision of quality healthcare?
	What factors contribute to your motivation?	How important is healthcare workers motivation in providing quality healthcare and productivity? If yes, in what way? Are there any non-monetary factors?
Health systems status and strengthening	What is the role of health, happiness, and motivation of healthcare workers in the provision of quality health services?	What is the role of health, happiness, and motivation of healthcare workers in the performance of the health system? In your opinion, what is the role of healthcare workers health, happiness, and motivation in the responsiveness to non-medical patients expectations (e.g., waiting time, confidentiality of patient information, courteous)?
	Do healthcare workers have healthcare insurance covers?	Does it insure healthcare workers and their family? Does it apply to public, mission and private-for-profit facilities?
	In relation to the health workforce, what are the key challenges within the hospital (possibly in the current health system)?	In your opinion, how could some of these challenges be addressed?
	How are healthcare workers health, happiness and motivation promoted in policy development and implementation?	What policies are in place to enhance the health, happiness, and motivation of healthcare workers in the health system? Are you aware if healthcare workers health, happiness and motivation policies are reflected in the national health policy and strategic plan? Do you think that the ministry of health should develop and institutionalize healthcare workers happiness and motivation program?
Conclusion of the interview	Is there anything you would like to add in relation to the topic we have discussed? (comments, challenges, or suggestions).	
